# Identification of potential biomarkers for oral squamous cell carcinoma through multi-cohort bioinformatic analysis

**DOI:** 10.1186/s43046-026-00392-5

**Published:** 2026-08-11

**Authors:** Duanreiliu Kamei, Simran Kaur, Anjali Priya, Akshay Bansal, Aarti Yadav, Ashwini Kumar Ray, Yamini Agrawal

**Affiliations:** 1 Department of Botany, University of Delhi, New Delhi, India; 2 Department of Environmental Studies, University of Delhi, New Delhi, India; 3 Department of Periodontology, Inderprastha Dental College & Hospital, Ghaziabad, India; 4 Lady Irwin College, University of Delhi, New Delhi, India

**Keywords:** EGFR, FN1, IL6, PTPRC, STAT1

## Abstract

**Background:**

Oral squamous cell carcinoma (OSCC) arises in the context of diverse etiological exposures, such as tobacco, alcohol, areca nut use and viral infections. This etiological heterogeneity drives distinct molecular alterations, contributing to tumor complexity and significant challenges in identifying robust, clinically applicable biomarkers.

**Methods:**

To resolve this, we employed an integrative bioinformatics approach, analyzing five gene expression datasets retrieved from the GEO repository, which encompass heterogenous clinical samples with diverse clinical stages of OSCC Differentially expressed genes (DEGs) were identified using stringent thresholds (|LogFC|≥ 1.5 to 3.0; *p* < 0.05), followed by GO and KEGG pathway enrichment analysis. A protein–protein interaction (PPI) connectome was generated, leading to identification of key hub genes using five topological properties. The biological relevance of hub genes was validated via GEPIA, HPA, immune infiltration analysis, and miRNet.

**Results:**

Screening of datasets provided 1764 DEGs. Enrichment analysis revealed dysregulation in immune response, metabolic processes, remodeling of the extracellular matrix, and inflammatory signaling. Five hub genes, *EGFR, FN1, IL6, STAT1*, and *PTPRC*, emerged as central regulators with distinct expression-survival profiles and associations with immune infiltration patterns. Notably, *STAT1* and *IL6* demonstrated context-dependent behavior, reflecting both tumor-intrinsic and microenvironment-derived expression patterns.

**Conclusion:**

This study highlights five key genes with potential diagnostic, prognostic, and therapeutic relevance in OSCC, emphasizing their consistency across clinically heterogeneous patient cohorts.

**Impact:**

These results extend a mechanistic understanding of OSCC pathobiology, thus promising leads towards the development of clinically robust biomarkers.

**Supplementary Information:**

The online version contains supplementary material available at https://doi.org/10.1186/s43046-026-00392-5.

## Introduction

Oral cancer remains a significant health concern globally, particularly in developing countries, where it causes about 389 000 fresh cases and 180 000 mortalities annually, as mentioned in the GLOBOCAN report, 2022 [8]. In the year 2020, India alone reported around 130 000 new cases and 75 000 patient deaths [50]. Oral squamous cell carcinoma (OSCC) constitutes approximately 95% of all head and neck malignancies [34] with a five-year survival rate of < 50%, making it the common histological form [2, 10]. OSCC most frequently arises on the tongue’s lateral border, the mouth’s floor, and the lower lip, although it can originate at various anatomical sites within the oral cavity [20, 42, 59].

The oral cavity harbors a distinct microenvironment shaped by its unique tissue architecture, exposure to diverse microbial communities, saliva composition, epithelial turnover, and frequent contact with external carcinogens. These site-specific features not only influence the pathogenesis of OSCC but also differentiate it from carcinomas arising in other tissues [35]. A major obstacle in understanding and managing OSCC is its pronounced tumor heterogeneity, which spans histological, molecular, anatomical, ethnic, and microenvironmental dimensions, creating significant challenges for biomarker identification, early diagnosis, and therapeutic intervention [60]. Intra-tumoral heterogeneity (ITH) arises from a combination of genetic, epigenetic, metabolic, and immunological alterations influenced by both intrinsic factors and extrinsic exposures. Key environmental risk factors contribute significantly to disease onset and progression, including tobacco use, areca nut chewing, alcohol consumption, exposure to radiation, Human papillomavirus (HPV) infection, and poor oral hygiene [48, 49]. Among these, intake of alcohol and tobacco remain most prominent risk contributors, together accounting for approximately 75% of all OSCC cases [21]. While HPV plays a critical role in OSCC (particularly those arising from the base of the tongue and tonsils), its etiological relevance in OSCC appears to be less prominent and regionally variable [21].

However, despite technological advances in molecular profiling, the complex and multifactorial nature of OSCC continues to limit the current biomarker’s sensitivity, specificity, and clinical utility. As OSCC primarily spreads via the lymphatic system, nodal metastasis significantly worsens prognosis [27]. Cervical lymph node metastasis is the most decisive prognostic factor in OSCC [1] and is associated with up to a 50% reduction in overall survival, particularly when nodal burden is high [25] and is considered a key determinant of OSCC related mortality [27]. Beyond regional nodes, distant metastasis severely damages long-term outcomes,patients with newly diagnosed metastatic tongue squamous cell carcinoma suffer a median survival time of only 4.0 months [9]. Metastasis may represent an adaptive escape mechanism of tumor cells from a hostile microenvironment, characterized by hypoxia, nutrient deprivation, inflammation, and immune surveillance [30]. Current imaging methods often fail to detect micrometastases, underscoring the urgent need for molecular biomarkers to enable early detection [27].

To address these challenges, this study employs a comprehensive bioinformatics strategy that integrates multiple gene expression datasets from the Gene Expression Omnibus (GEO) database, capturing diverse clinical stages of primary tumor development, metastasis, and recurrence, to identify consistent differentially expressed genes (DEGs) between OSCC and normal tissues across heterogeneous patient samples. By constructing protein–protein interaction (PPI) networks and applying multiple topological algorithms, the study aims to identify central molecular markers in OSCC. This integrative, systems-level approach provides biologically relevant and clinically actionable insights into the molecular heterogeneity of OSCC.

## Materials and methods

**Fig. 1:**
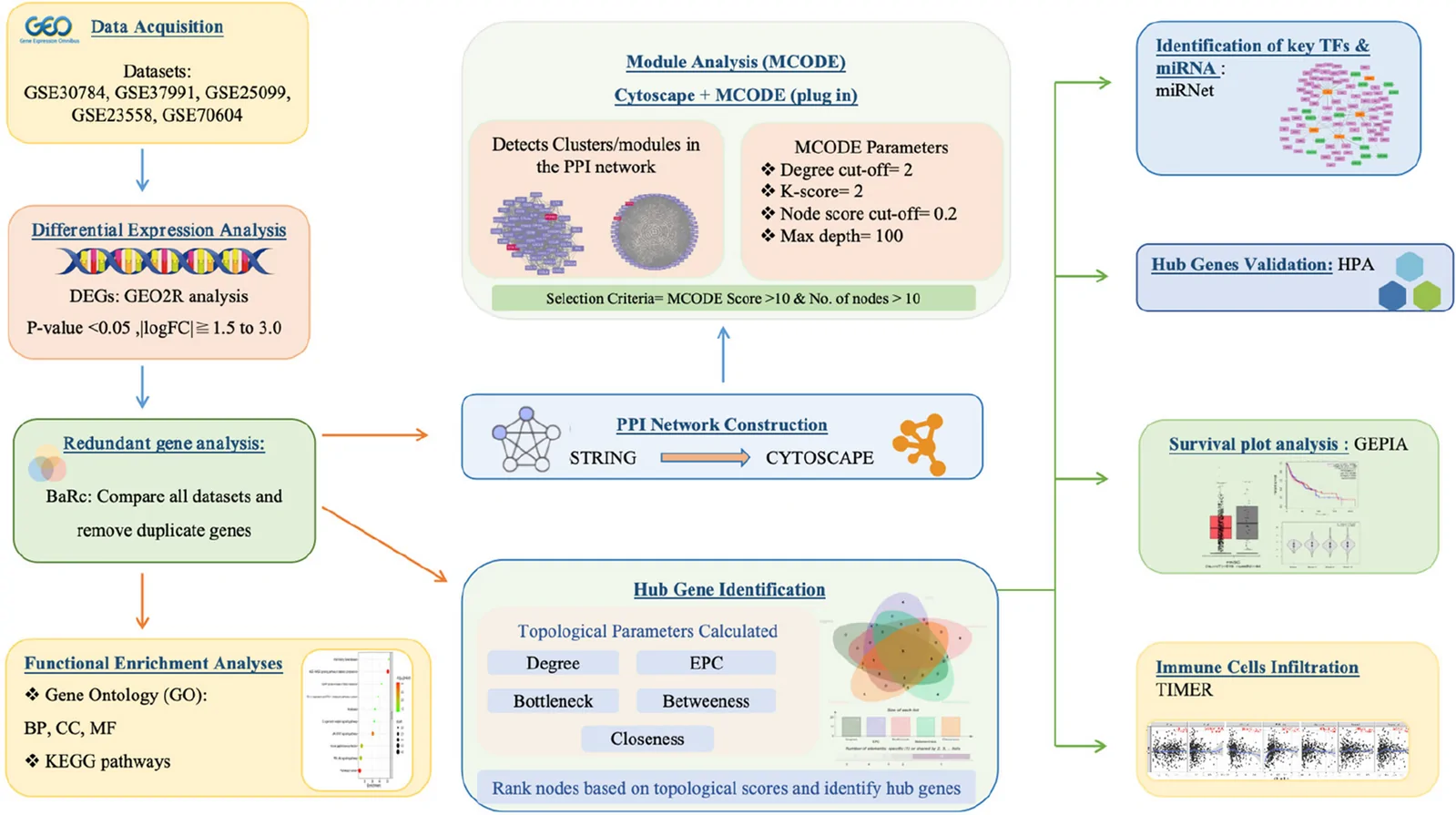
Schematic workflow of the methodology employed in this study

A schematic workflow of the methodology employed in the study is given in Fig. 1.

### Data collection

Gene expression data related to OSCC were retrieved using National Center for Biotechnology Information (NCBI) GEO repository (https://www.ncbi.nlm.nih.gov/geo/, accessed on 25th April, 2025), using “OSCC” as the search keyword [17]. Based on relevance and the use of microarray-based expression profiling, the following five datasets were selected for further analysis: GSE70604, comprising OSCC samples with and without lymph node metastasis, allowing exploration of metastasis-associated gene expression signatures, and GSE30784, GSE37991, GSE25099, and GSE23558, which were selected to ensure a broad representation of OSCC patient profiles across distinct clinical stages including primary tumorigenesis, recurrence, and metastasis. Detailed information on these datasets, including the number of controls and cases, is provided in Table S1.

### Differential expression profiling

The Geo2R tool embedded in the GEO database was employed to screen the DEGs among the normal and OSCC cells in the study datasets. Different log fold change (logFC) was used for each dataset for the selection of DEGs in the GEO datasets, and an adjusted p-value less than 0.05 was used as cut off for screening criterion [7]. Gene expression data in the GSE25099 dataset were originally annotated with Ref-seq transcript IDs (e.g., NM_015848). Dataset-specific logFC thresholds (1.5–3.0) were selected to obtain a comparable number of highly significant DEGs while maintaining an adjusted *p*-value < 0.05. These were mapped to official gene symbols using Genecards (https://www.genecards.org/) [40], and NCBI Gene databases for downstream analysis. All the datasets were compared using the bioinformatics and research computing (BaRC): redundant list analyses tool (http://barc.wi.mit.edu/tools/), to remove the duplicate genes.

### Biological function and pathway enrichment analysis for DEGs

The OSCC DEGs, both upregulated and downregulated, identified from the GEO database were analyzed for GO and KEGG enrichment pathways employing the Database for Annotation, Visualization and Integrated Discovery (DAVID) [39]. The studies were conducted to investigate their biological functions and subsequently pathways. The GO annotation findings categorize gene function into three groups: Biological process (BP), Cellular component (CC), and Molecular functions (MF). Upregulated and downregulated gene sets were separately analyzed by uploading the official gene IDs under the “functional annotation tool” of DAVID and mapping them against the background genes of *Homo sapiens*. Charts for GO term BP, CC, MF and KEGG pathways were generated using a threshold of Benjamini corrected *p-*value less than 0.05 for statistical significance. The top 20 enriched terms from each category (from highest to lowest) were visualized using SRplot (https://www.bioinformatics.com.cn/en) [43].

### OSCC PPI network mapping and analysis

The construction of an OSCC PPI network, which is a map of physical associations between proteins within a cell or organism, involves analyzing how different proteins interact with each other. The Search Tool for the Retrieval of Interacting Genes (STRING) repository (https://string-db.org/) [41] was utilized to obtain protein–protein interactions data by uploading the up- and downregulated DEGs. The physical interaction data of STRING were imported and used for the construction of the OSCC PPI network using the Cytoscape v3.10.3 visualization and analysis software (https://cytoscape.org/) [38].

### Identification of hub genes

The network topology indices of the constructed interactome were computed using CytoHubba (https://apps.cytoscape.org/apps/cytoHubba) [15] plugin network analyzer in Cytoscape 3.10.3 to identify the hub genes while treating the network as a unidirectional network. The tool predicts and explores important hubs/nodes and sub-networks in a given network by providing topological parameters, including Edge Percolated Component (EPC), degree, maximum neighborhood component (MNC), maximal clique centrality (MCC), density of maximum neighborhood component (DMNC), clustering coefficient (CC), EcCentricity, bottleneck, closeness, betweenness, radiality, and stress.

Hub genes or the highest degree nodes are considered the critical regulators and crucial agents in many signaling pathways; they also preserve the network topological features, stability, and self-organization [32]. The hub genes were identified considering five topological properties, which include degree, EPC, bottleneck, betweenness centrality and closeness, which represent the node with the highest number of connections, critical nodes that preserve network robustness, and nodes that are crucial for network connectivity and information flow. The tool jveen (https://jvenn.toulouse.inrae.fr/app/index.html) was used to construct a Venn diagram to overlap the genes of the five topological attributes [6].

### Module analysis

The Molecular complex detection (MCODE) (https://apps.cytoscape.org/apps/mcode) [3] plug-in of Cytoscape finds clusters/modules, which are a group of highly interconnected nodes within a larger network. MCODE version 2.0.3 is used to define functional modules in the PPI network. The following default MCODE parameters were used for network scoring and cluster finding to detect highly connected regions: “Degree cut-off = 2”, “K-score = 2”, “node score cut-off = 0.2”, and “max depth = 100”. Modules were screened according to the following criteria: MCODE score > 10 and number of nodes > 10 [57].

### Survival analysis of identified hub genes

The gene expression profiling interactive analysis (GEPIA) (http://gepia.cancer-pku.cn/) [44] bioinformatics tool was used in this study to assess whether the expression levels of the key genes were associated with the survival of OSCC patients. The overall survival (OS) analysis was performed using the web server GEPIA based on the head and neck squamous cancer (HNSC) available on the cancer genome atlas (TCGA) and genotype tissue expression (GTEx) data. The median expression level was used to divide the patients with OSCC into high and low expression groups. GEPIA also uses the log-rank (Mantel–Cox) test for hypothesis evaluation. The Cox proportional hazard ratio (HR) and 95% confidence interval (CI) were included in the survival plot to assess statistical significance (*p* < 0.05). The survival curves were plotted with time expressed over months. Box plot and pathological stage plot were used to validate the important hub genes.

### Identification of miRNA and transcription factors (TFs) against key genes

The dysregulation of miRNA and TFs has been associated with the progression of numerous diseases including OSCC [19, 24]. In this study, miRNET (https://www.mirnet.ca/) [12], a computational biology tool, was used to analyze miRNAs and their associated transcription factors for the selected key genes. A network of the predicted miRNA-TFs and genes obtained from miRNet was imported into Cytoscape for visualization.

### Validation of hub genes at the protein level

The Human Protein Atlas (HPA) (https://www.proteinatlas.org), a publicly available repository for a comprehensive human proteome atlas using antibody-based proteomics, transcriptomics, and other omics technologies. It also gives detailed protein expression patterns in diseased and normal tissues. It was employed to analyze the translational expression patterns of the five hub genes [45].

### Immune infiltration profiling

To evaluate the correlation between the selected key regulatory genes [epidermal growth factor receptor (EGFR), interleukin 6 (IL6), signal transducer and activator of transcription 1 (STAT1), fibronectin 1 (FN1), and protein tyrosine phosphatase receptor type C (PTPRC)] and immune cells (B cells, CD4 + T cells, CD8 + T cells, neutrophils, macrophages, and dendritic cells) infiltration in patients with HNSC, a Tumor Immune Estimation Resource (TIMER 2.0) (https://cistrome.shinyapps.io/timer/) repository was utilized [26]. Since OSCC accounts for ~ 95% of all HNSC cases and a specific OSCC category is not included in TCGA database, similar to other studies, TCGA − HNSC data were obtained and used in this analysis [51].

## Results

### Analysis of differentially expressed genes

Genes with an adjusted p value less than 0.05 and fold change threshold ranging from |logFC| ≧ 1.5 to 3.0 were considered significantly differentially expressed. From an initial pool of 1,490 overexpressed and 1,088 underexpressed genes, duplicate entries were systematically filtered out using the Bioinformatics and Research Computing (BaRC) redundant list analysis tool. Finally, 1764 significant and unique genes (comprising 1,015 upregulated and 749 downregulated genes) were retained for further analysis. The list of up- and downregulated genes along with their respective fold change of the datasets is described in Table 1.

**Table 1:** Differentially expressed genes from OSCC datasets used in the study

Datasets	Up regulated	Down regulated	logFC Threshold	*p* value
GSE30784	305	229	|logFC|≧2.5	< 0.05
GSE37991	346	143	|logFC|≧2	< 0.05
GSE70604	237	365	|logFC|≧2.5	< 0.05
GSE23558	399	136	|logFC|≧3	< 0.05
GSE25099	203	215	|logFC|≧1.5	< 0.05

### Biological functional and pathway enrichment analysis of DEGs

**Fig. 2:**
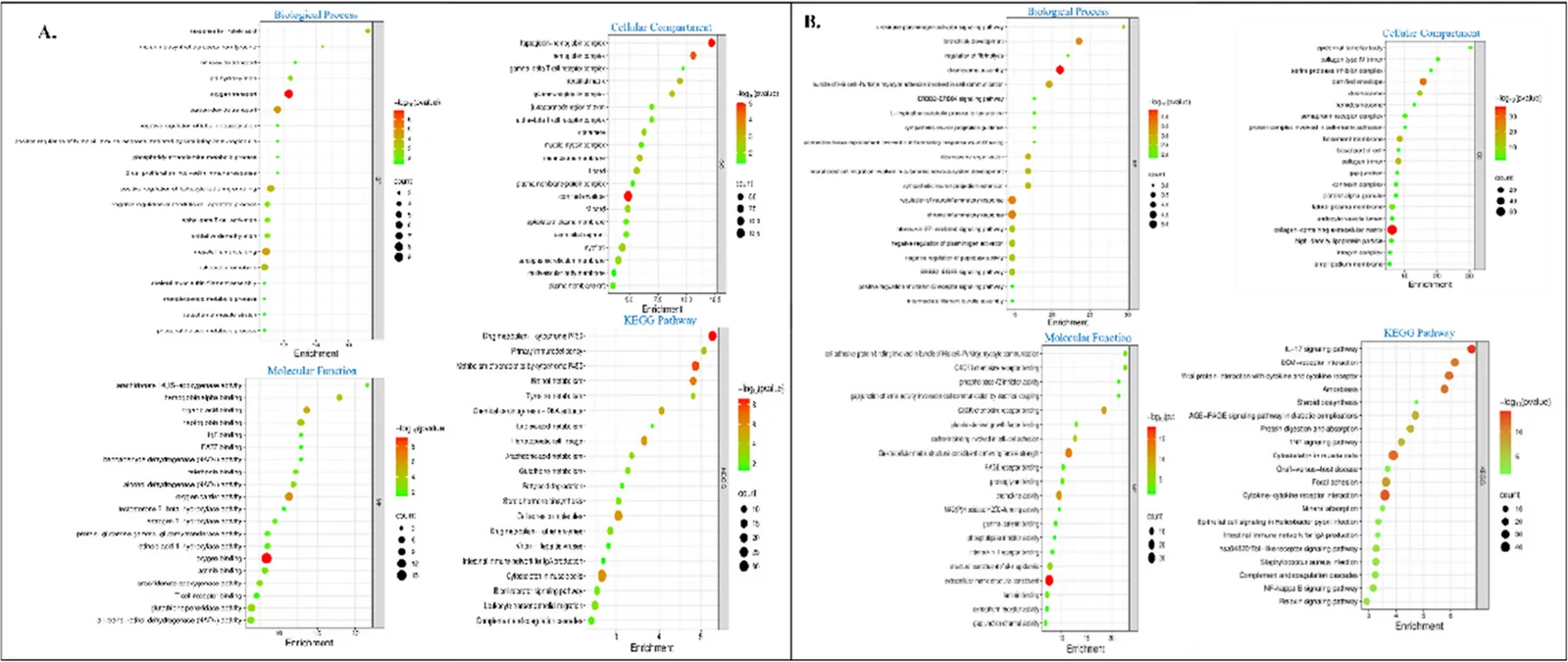
Functional enrichment in OSCC: **A** upregulated DEGs; **B** downregulated DEGs

The upregulated and downregulated genes were functionally annotated using the DAVID database, and the GO and KEGG enrichment cascades were visualized using SRplot. The bubble plot in Fig. 2A illustrates the enriched top 20 GO terms and KEGG cascade for the upregulated genes. GO BP enrichment revealed that the upregulated genes were significantly involved in metabolic and transport pathways, including response to linoleic acid, nitric oxide transport, lipid hydroxylation, oxygen and carbon-dioxide transport, and phosphatidylethanolamine metabolic process. They were also strongly associated in immune-related processes, such as positive regulation of humoral immune response, B cell proliferation, and leukocyte chemotaxis. In addition, the cornified envelope, IgG immunoglobulin complex, and plasma membrane protein complex contain topmost upregulated genes. As for MF, the upregulated DEGs were significantly associated with oxygen binding and carrier activities, hemoglobin alpha binding, glutathione peroxidase activity, estrogen hydrolase activity, and alcohol dehydrogenase activity, among others. KEGG pathway enrichment analysis indicated that the differentially upregulated genes were mainly involved in pathways such as drug metabolism-cytochrome P450, metabolism of xenobiotics by cytochrome P450, primary immunodeficiency, glutathione, linoleic acid metabolism, chemical carcinogenesis-DNA adducts and steroid hormone biosynthesis.

The bubble plot in Fig. 2B illustrates the top enriched GO terms and KEGG pathways for differentially downregulated genes. In the BP category, downregulated genes primarily enriched pathways such as the regulation of fibrinolysis, ERBB2–ERBB4 signaling pathway, interleukin-27-mediated signaling pathway, and ERBB2–*EGFR* signaling pathway. In the CC category, these genes were mainly located in collagen-containing extracellular matrix, cornified envelope, basement membrane, desmosome and collagen trimer. Gene Ontology MF analysis revealed that the downregulated genes were significantly correlated to extracellular matrix structural constituent, structural constituent of skin epidermis, CXCR chemokine receptor binding, cadherin binding involved in cell–cell adhesion and others. KEGG pathway enrichment showed that the downregulated DEGs were involved in pathways such as IL-17 signaling, cytokine-cytokine receptor interaction, TNF signaling pathway, ECM-receptor interaction, and focal adhesion, etc*.*

### Protein–protein interaction network mapping and analysis

A set of 1,764 up- and downregulated DEGs were submitted to the STRING database for the construction of a PPI network. The physical interaction data obtained from STRING were imported into Cytoscape for network analysis. The resulting PPI network consisted of 1,524 nodes and 18,821 edges after the removal of isolated nodes and duplicate edges respectively, representing the OSCC system.

### Identification of Hub genes

**Fig. 3:**
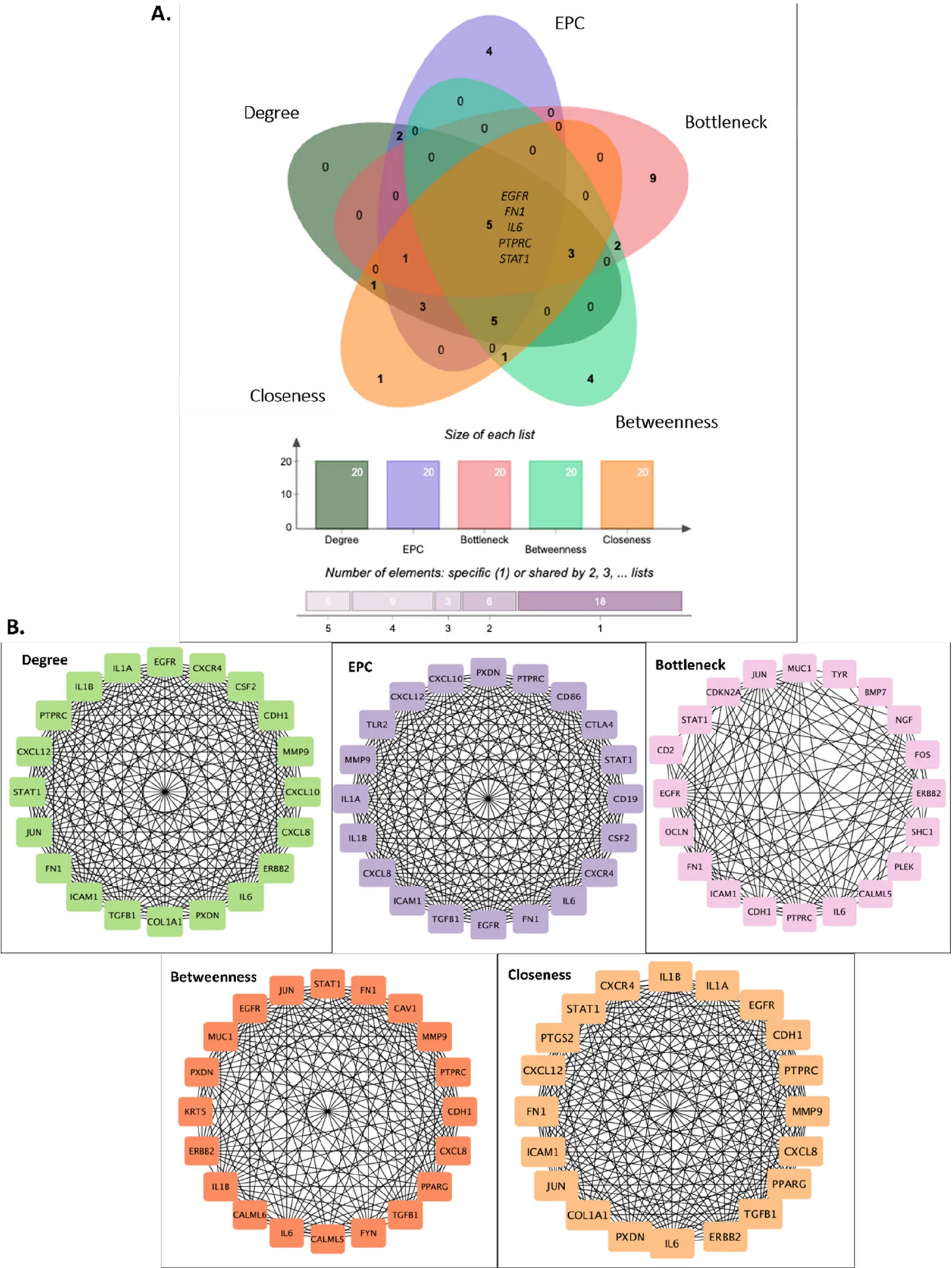
Identification of hub genes using network topological properties: **A** Venn diagram showing the number of common genes among the top 20 genes topological properties, i.e., degree, EPC, bottleneck, betweenness, and closeness centrality; **B** Regulatory network of the top 20 topological properties of OSCC

Further network analysis using the CytoHubba plugin in Cytoscape revealed hub genes using five topological properties: degree, EPC, bottleneck, betweenness centrality, and closeness. The top 20 genes sorted in decreasing order for each property were obtained and are listed in Table S2. After examining the topological properties and correlating them with the 20 hub genes, epidermal growth factor receptor (EGFR), interleukin 6 (IL6), signal transducer and activator of transcription 1 (STAT1), fibronectin 1 (FN1), and protein tyrosine phosphatase receptor type C (PTPRC) which are common to all five topological attributes, were considered as the key regulatory genes. A Venn diagram illustrating the overlapping genes among the top 20 ranked genes identified by the five topological attributes along with the regulatory network of each attribute is shown in Fig. 3.

### Module analysis

MCODE plugin was performed to assess the biological significance and network positioning of the identified hub genes in densely connected modules in the PPI networks. Modules were screened according to the following criteria: MCODE score > 10 and number of nodes > 10 [57]. A total of 41 modules were identified, out of which 3 modules having MCODE score more than 10 and nodes > 10 were considered significant and filtered from the PPI network. Among the top 3 results, module 1 had 50 nodes and 927 edges having a score 37.837, module 2 had 84 nodes and 1240 edges with an MCODE score 29.880, and lastly, module 3 had 108 nodes and 982 edges having a MCODE score of 18.355. Notably, all five hub genes were located within the top modules: two hub genes were localized in module 1 (score: 37.837), two in module 2 (score: 29.880), and one in module 3 (score: 18.355) as shown in Figure S1. The presence of hub genes in the highest-scoring and most densely connected modules supports their functional significance and centrality within the OSCC-associated protein interaction network.

### Survival plot analysis of hub genes

**Fig. 4:**
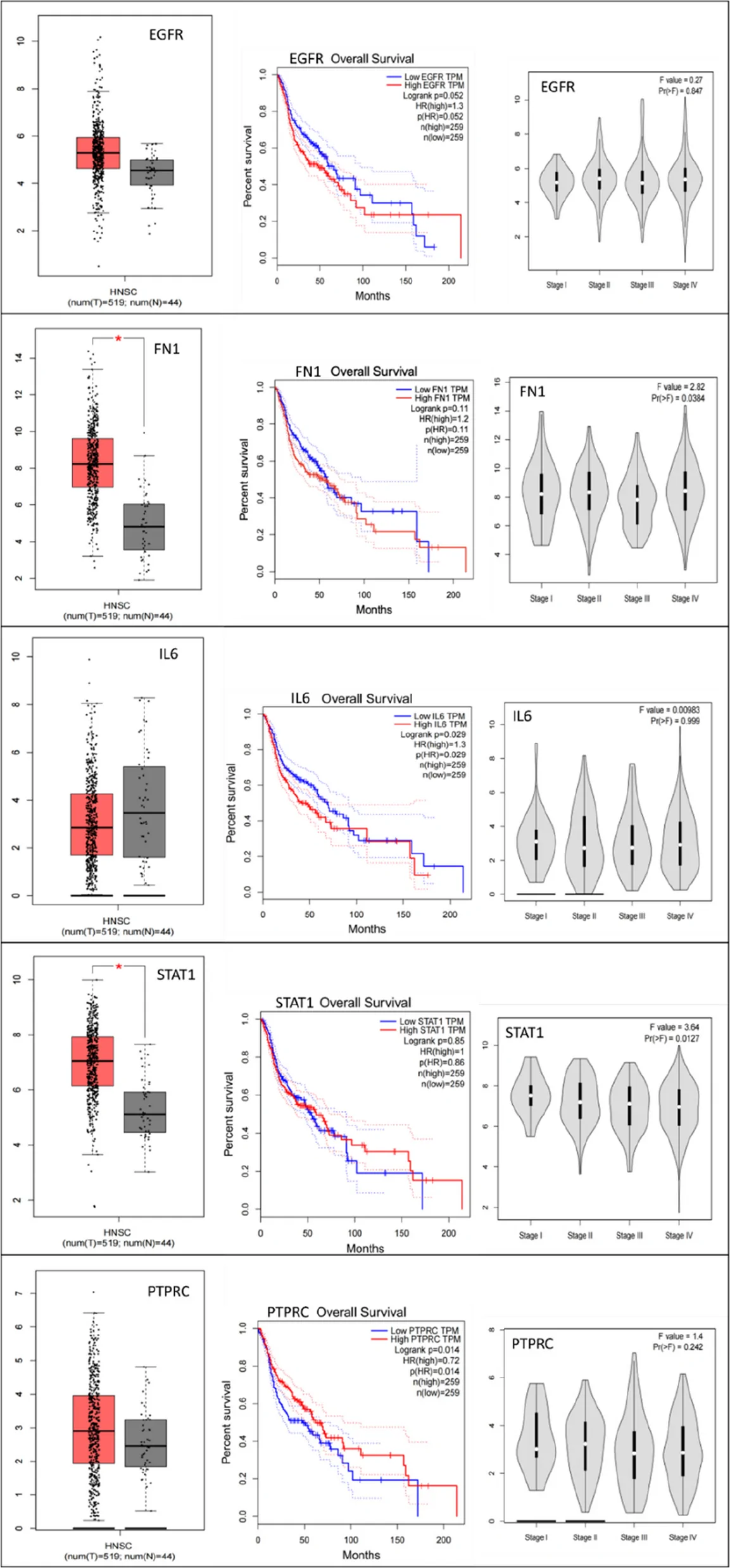
Comparative gene expression, overall survival and pathological stage profiling of hub genes. (Normal: gray; Tumor: red; *p* < 0.05)

Using GEPIA, an online tool for gene expression profiling, the overall survival of the identified hub genes (*EGFR*, *FN1*, *IL6*, *STAT1*, and *PTPRC*) were plotted as shown in Fig. 4. *EGFR* is significantly overexpressed in tumor tissue relative to normal tissue as shown in the HNSC dataset. The analysis of overall survival (OS) indicates that high expression of *EGFR* in patients had a poorer OS. Further, this suggests an increased risk of death compared with those with low expression. Violin plot analysis for the expression of *EGFR* across different pathological stages (I-IV) of HNSC, indicating F value = 0.27, implying no significant difference in expression across different stages. In the subsequent panel, *FN1* is shown to be markedly overexpressed in tumor specimens relative to normal tissues in the HNSC dataset. The OS analysis indicated that elevated *FN1* expression in patients was correlated with reduced OS. The violin plot revealed that *FN1* expression is significantly varied with F value = 2.82 indicating its association with disease progression. Next, the expression of *IL6* in the tumor tissue is found to be significantly lower than in normal tissue in the HNSC dataset, suggesting that *IL6* may be downregulated in cancer. However, the OS analysis of *IL6* indicated that elevated *IL6* expression is significantly associated with poorer survival outcomes despite the lack of differential expression. Additionally, the violin plot shows no significant variation of *IL6* expression across different pathological stages of cancer, implying that *IL6* level is independent of tumor progression. The expression of *STAT1* is substantially overexpressed in the HNSC than the normal tissue. Also, the OS shows that *STAT1* expression levels do not significantly influence OS in this dataset with HR value 1. Thus, *STAT1* does not appear to be a prognostic biomarker for OS in this context. Violin plot analysis for *STAT1* demonstrated a statistically significant variation in *STAT1* expression across pathological stages (I–IV), indicating that *STAT1* expression may be influenced by tumor progression despite not impacting the OS. Further, elevated *PTPRC* expression in tumor tissue is seen compared with normal tissue in the HNSC dataset. The OS analysis revealed that *PTPRC* overexpression is associated with better survival than the reduced expression. Moreover, there is not a significant difference in *PTPRC* expression across different cancer stages (I-IV).

### Identification of miRNA and TFs against key genes

**Fig. 5:**
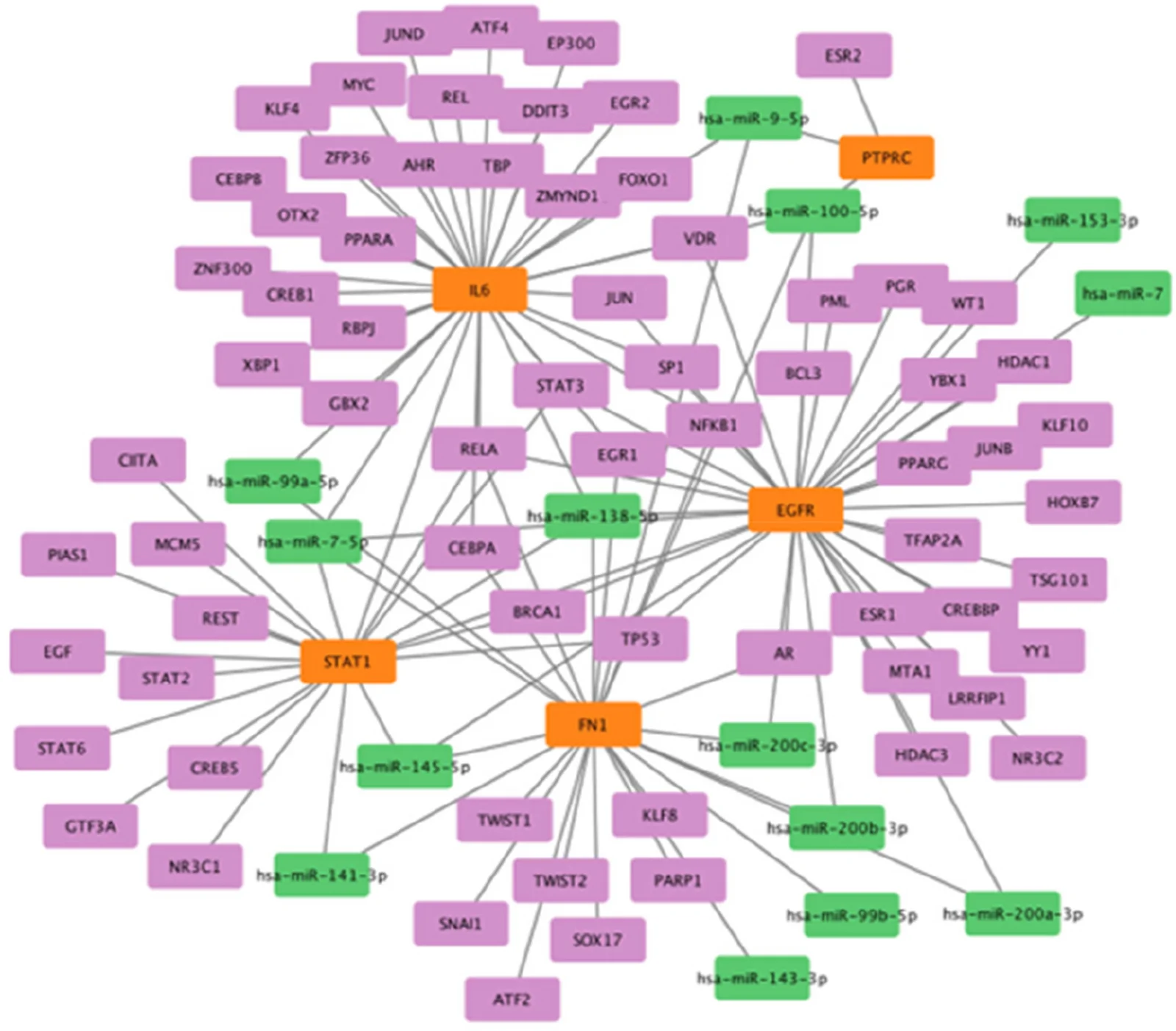
The key regulator genes-miRNA-TF regulatory network of OSCC-specific miRNAs for *EGFR*, *IL6*, *FN1*, *STAT1*, and *PTPRC* constructed using the miRNet database. (Orange: hub genes, green: transcription factors, purple: miRNAs)

An integrated online tool, miRNet, was used to create a probable network of miRNA and TFs of the five hub genes *EGFR*, *FN1*, *IL6*, *STAT1*, and *PTPRC*. A total of 13 miRNAs and 69 TFs were recovered from the network as depicted in Fig. 5.

### Validation of hub genes at the protein level

**Fig. 6:**
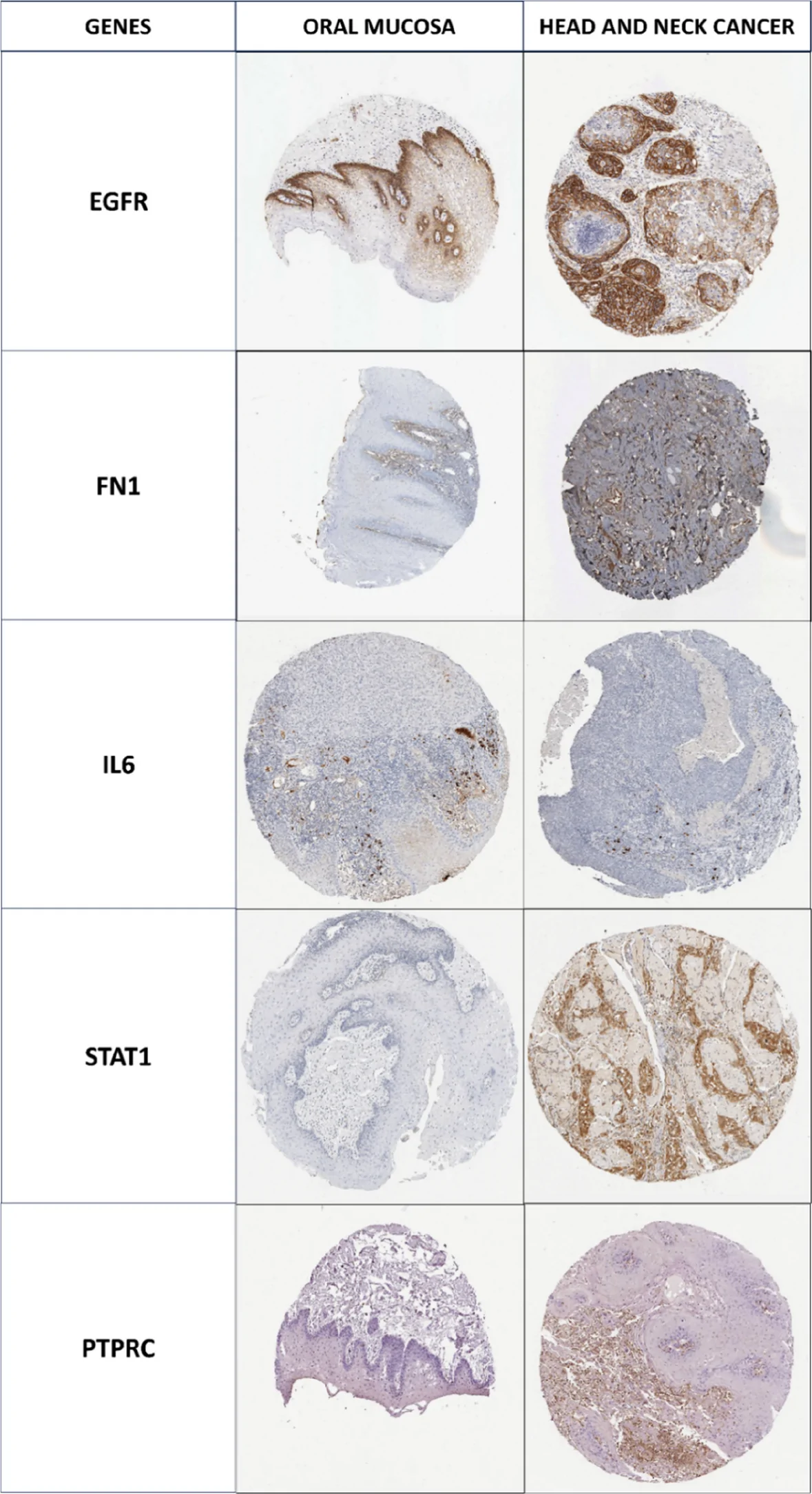
Representative immunohistochemical images of *EGFR*, *FN1*, *IL6*, *STAT1*, and *PTPRC* in normal oral tissue and HNSC tissues (Human Protein Atlas database)

High *EGFR* immunoexpression was observed in both normal and carcinoma tissues. *FN1* was absent or low in normal tissue but showed positive expression in carcinoma tissue. *IL6* was expressed in normal tissue but not in carcinoma tissue. *STAT1* exhibited high expression in carcinoma tissue but low expression was observed in non-tumor tissue. Expression of *PTPRC* was negative in both normal and carcinoma tissues as shown in Fig. 6 and Table S3. The selected hub genes were validated using the HPA, and the results were consistent with the findings of the survival plot analysis.

### Immune infiltration profiling

**Fig. 7:**
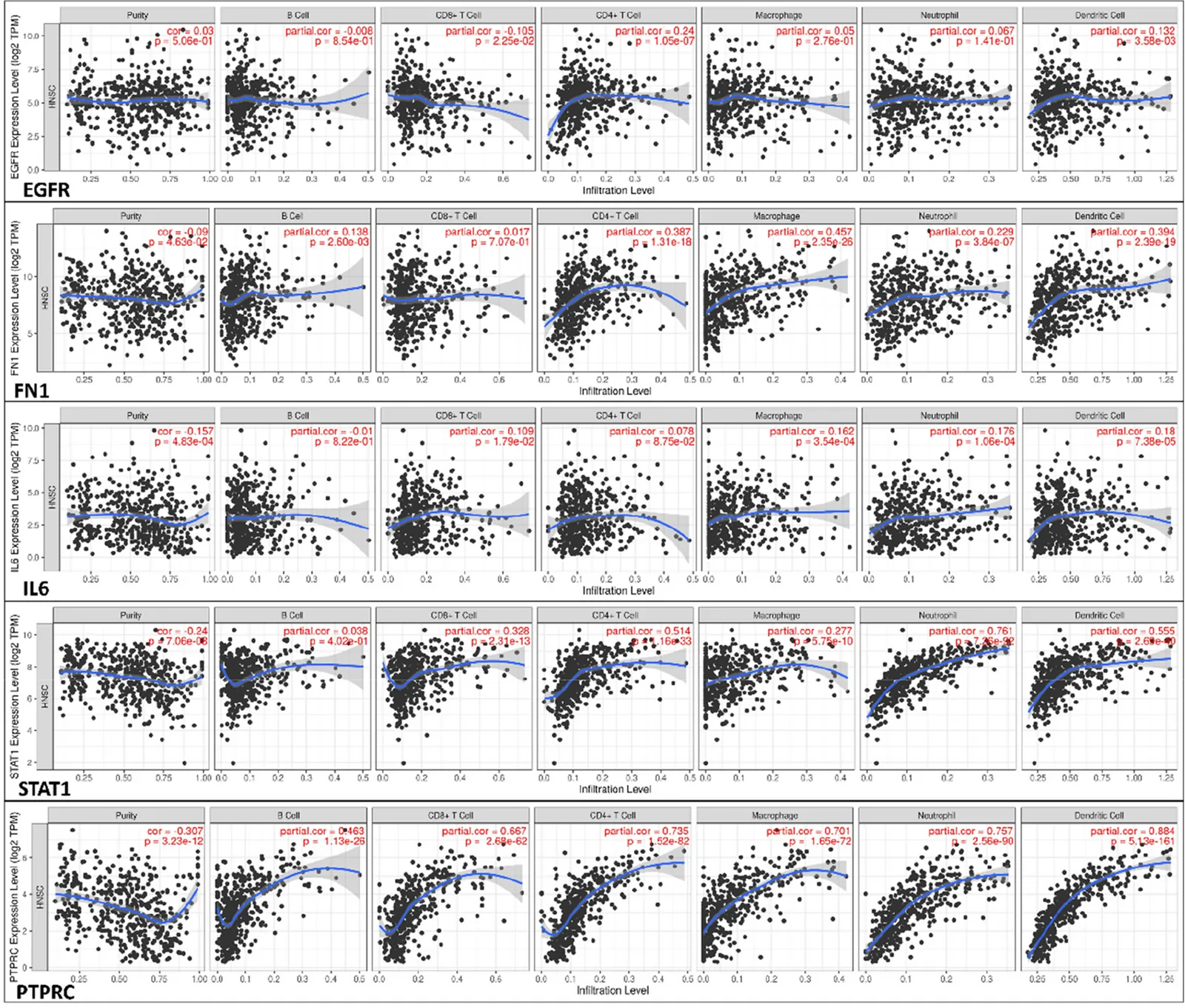
Correlation between *EGFR*, *FN1*, *IL6*, *STAT1*, and *PTPRC* gene expression and immune cell infiltrating level derived from the TIMER 2.0 database. The X-axis represents the levels of infiltration of a specific type of immune cell, whereas the Y-axis represents the gene expression level (log2 TPM). Partial Cor. > 0 = positive correlation, and Partial Cor. < 0 = negative correlation

Using the TIMER database, the correlation between mRNA expression levels of all genes and the immune cell infiltration in HNSCs was analyzed. The results revealed that *FN1*, *IL6*, *STAT1*, and *PTPRC* except for *EGFR*, were negatively related to tumor purity, suggesting that these four genes were produced in the microenvironment and not the tumor cells themselves, while *EGFR* is tumor cell derived. The mRNA expression levels of *EGFR*, *FN1*, *IL6*, *STAT1*, and *PTPRC* showed a significant positive correlation associated with the infiltrating levels of CD4 + cells, neutrophils, macrophages, and dendritic cells, highlighting their role in shaping the tumor immune microenvironment. Furthermore, *FN1*, *IL6*, *STAT1*, and *PTPRC* were positively associated with CD8 + T cell infiltration, whereas *EGFR* showed an inverse relationship, supporting its role in tumor evasion and progression. Expression of *FN1*, *STAT1*, and *PTPRC* also positively correlated with B Cells infiltration, suggesting upregulation in the presence of B Cells, whereas *EGFR* and *IL6* showed a negative correlation with it. These results underscore the complex interplay between tumor cells and the immune system in OSCC, with distinct patterns of immune interaction observed across the identified hub genes. Partial correlation among the hub genes and the immune cell infiltrates are described in Fig. 7 and Table S4.

## Discussion

OSCC is a biologically intricate cancer characterized by extensive intra-tumoral heterogeneity (ITH), which contributes to diagnostic challenges, variable treatment responses, and poor prognostic outcomes [33]. Despite advances in molecular diagnostics, the multifactor nature of OSCC biology affects the sensitivity and specificity of existing biomarkers. ITH arises from cumulative genetic, epigenetic, metabolic, and immune-related alterations, influenced by both intrinsic factors and extrinsic exposures such as tobacco, areca nut, alcohol, radiation, HPV, and poor oral hygiene [48, 49]. With these challenges in mind, this study employed an integrative bioinformatics strategy to identify consistent and functionally significant biomarkers that account for OSCC’s heterogeneity across different clinical factors.

Five microarray datasets from GEO were analyzed using a standardized differential gene expression approach to mitigate dataset-specific biases and enhance biological relevance. The use of stringent thresholds for fold change (|logFC|≧1.5 to 3.0) and *p*-values (< 0.05) ensured high-confidence DEG selection. The identification of 1764 DEGs, (1015 upregulated and 749 downregulated) emphasizes transcriptional reprogramming in OSCC. Cross-comparison and removal of redundant entries enhanced consistency and minimized analytical noise.

Enrichment analysis studies with GO and KEGG with DAVID database showed significant enrichment of upregulated DEGs in drug metabolism (cytochrome P450), metabolism of glutathione, immune modulation, and oxygen transport. These findings align with the altered metabolic and redox status in OSCC, which likely contributes to its resistance to adverse microenvironmental conditions, as discussed earlier [13, 52, 53]. Conversely, downregulated DEGs were enriched in IL-17 and TNF signaling, ECM-receptor interactions, and focal adhesion processes, reflecting disruptions in structural integrity, inflammation, and immune regulation. Dysregulation of cell–matrix adhesion in HNSC promotes invasion of tumor and metastasis via regulation of matrix metalloproteinase expression [16, 36, 56]. These findings highlight distinct biological themes underlying OSCC progression: metabolic activation, immune evasion, and matrix remodeling. By constructing a comprehensive PPI network (1524 nodes, 18,821 edges) and applying five topological algorithms via CytoHubba, we identified five hub genes: *EGFR*, *FN1*, *IL6*, *STAT1*, and *PTPRC*. These genes were consistently ranked across multiple centrality measures (Degree, EPC, Betweenness, Bottleneck, Closeness), suggesting their central roles in the biological network of OSCC. Additionally, MCODE clustering confirmed the localization of these hub genes within the top 3 most densely connected modules, reinforcing their topological and biological importance.

*EGFR* is a well-characterized proto-oncogene belonging to the receptor tyrosine kinase (RTK) family, known for its critical role in regulating cell growth, survival, and differentiation [47]. In OSCC, *EGFR* is commonly upregulated, leading to a poor prognosis and aggressive tumor behavior [22]. Our GEPIA analysis showed that *EGFR* is significantly overexpressed in OSCC tissues, with high expression linked to poorer OS, indicating its association with adverse outcomes. *EGFR* expression did not significantly differ across tumor stages, suggesting early and sustained dysregulation. High *EGFR* protein expression was confirmed in both normal and carcinoma tissues, implying that its oncogenic role depends more on activation status (e.g., ligand binding or phosphorylation) than expression levels alone. OSCC cells secrete epidermal growth factor (EGF) and transforming growth factor-α (TGF-α), leading to autocrine and paracrine activation of *EGFR* signaling. Its activation of MAPK, PI3K-AKT, and STAT signaling cascades supports its role in tumor proliferation, survival, and immune evasion [48, 49]. Notably, the TIMER analysis of *EGFR* showed a negative correlation with CD8 + T cells, which supports its role in immunosuppression and escape. For OSCC, *EGFR* may act as a promising biomarker for diagnostics, given its central involvement in tumor biology.

*FN1*, a major extracellular matrix protein, is frequently overexpressed in various cancers, including renal, thyroid, lung, and head and neck tumors [58]. In OSCC, *FN1* promotes tumor progression by activating several signaling cascades, such as FAK/ILK/ERK/PI3K/NF-κB, which upregulate MMP2 and MMP9, facilitating ECM degradation and cancer cell invasion [36]. Additionally, *FN1* alters the activity of tyrosine-protein kinase Met (c-Met), the receptor for hepatocyte growth factor (HGF), thereby activating MET-mediated PI3K/AKT signaling and promoting tumor growth and lymph node metastasis. *FN1* also acts in coordination with TGF-β to induce epithelial-mesenchymal transition (EMT), further enhancing the invasive and metastatic capabilities of OSCC cells [29]. GEPIA analysis in the present study confirmed the upregulation of *FN1* in OSCC and showed that higher expression levels are associated with reduced OS. Immune infiltration analysis using TIMER further revealed that *FN1* expression positively associated with the infiltration of CD8 + and CD4 + T cells, B cells, neutrophils, macrophages, and dendritic cells, suggesting a role in immune cell recruitment and tumor immune microenvironment modulation. This aligns with the findings of [52, 53] in gastric cancer, highlighting *FN1*’s consistent immunological relevance across cancer types. These findings support *FN1* as a potential diagnostic and therapeutic target in OSCC, linking ECM remodeling, oncogenic signaling, inflammation and immune evasion as key hallmarks that facilitate tumor initiation, progression, and metastasis in OSCC.

About 25% of malignancies are associated with chronic inflammation or infection [55]. Proinflammatory cytokines, particularly IL-6, IL-8, IL-12p70, and IL-17, are significantly involved in shaping the tumor microenvironment (TME), promoting cancer cell proliferation, invasion, and immune suppression [4]. Interleukin-6 (IL-6) is a multifunctional cytokine playing a pivotal role in OSCC progression via activating the JAK–STAT3 signaling cascade. IL-6, particularly when secreted by mesenchymal stem cells (MSCs) within the TME, induces EMT, enhancing tumor invasiveness and metastatic potential [28]. However, gene expression analysis in our study revealed reduced IL-6 mRNA levels in OSCC tumor tissues compared with normal tissues (*p* < 0.05). This apparent contradiction may be explained by the predominantly paracrine secretion of IL-6 from stromal cells, such as MSCs, rather than from tumor cells, emphasizing the necessity of comprehensively evaluating the TME rather than focusing solely on tumor cell expression profiles [26]. GEPIA analysis also revealed that patients with high *IL6* expression had significantly poorer survival, suggesting that *IL6*, although not tumor-derived, may exert its effects via stromal or immune cells. Moreover, cytokine *IL6* alteration may serve as a valuable biomarker for disease prognosis and monitoring.

Our GEPIA analysis revealed a significant *STAT1* overexpression in OSCC tissues (*p* < 0.05) compared with normal tissues in the HNSC cohort. However, despite this elevated expression, *STAT1* did not significantly influence overall survival, indicating that it may not serve as a conventional prognostic marker. Interestingly, *STAT1* expression varied significantly across tumor stages, suggesting a potential role in disease progression rather than prognosis. However, immunohistochemical data from the HPA showed moderate *STAT1* protein expression in both carcinoma and healthy tissues. Analysis using the TIMER database revealed that *STAT1* expression was positively associated with immune cell infiltration, including CD4 + and CD8 + T cells, B cells, neutrophils, macrophages, and dendritic cells. This suggests that *STAT1* may shape the immune microenvironment of tumors by promoting immune cell recruitment and activation. However, the role of *STAT1* in cancer is known to be highly context-dependent. *STAT1* can support tumor progression and immune evasion. For instance, it transcriptionally upregulates BST2, which activates the AKT/ERK1/2 pathway, promoting cell proliferation, invasion, and metastasis [37]. Additionally, IFN-γ-mediated activation of the JAK2/*STAT1*/STAT3 pathway induces PD-L1 expression, facilitating immune escape. IFN-γ also activates PKD3, leading to Ser727 phosphorylation of *STAT1* and STAT3, further enhancing their oncogenic potential [18]. Conversely, *STAT1* also possesses tumor-suppressive functions. It can activate pro-apoptotic genes, interact with p53, and inhibit MDM2, a negative regulator of p53, thereby enhancing p53-dependent apoptosis [46]. Notably, it was found that moderate levels of phosphorylated *STAT1* (p*STAT1*) were associated with improved survival in patients with OSCC treated with chemotherapy, indicating a predictive role in treatment response [31]. Therefore, *STAT1* has emerged as a context-dependent biomarker in OSCC playing both tumor-suppressive and tumor-promoting roles depending on the signaling context, phosphorylation status, and cellular environment. This dual nature indicates its relevance for use as a potential therapeutic target and predictive marker.

*PTPRC*, also known as CD45, encodes a transmembrane protein tyrosine phosphatase (PTP) that plays a pivotal role in regulating immune cell signaling. As a member of the PTP family, *PTPRC* modulates a range of cellular mechanisms, including growth of cell, mitosis, differentiation and transformation. *PTPRC* is crucial in regulating B and T cells antigen receptor signaling, by either directly interacting with the antigen receptor complexes or activating Src family kinases that are vital for downstream signaling pathways. It also functions as a negative regulator of cytokine receptor signaling by inhibiting JAK kinases [5]*.* Multiple alternatively spliced isoforms of *PTPRC* have been identified, among which CD45RO + T-cells have received considerable attention in the context of tumor immunology. CD45RO + T-cells, which represent memory T-cell subsets, are frequently enriched in the TME of various solid tumors. These cells are capable of recognizing and eliminating residual tumor cells, thereby improving the survival rate [23]. Earlier studies have demonstrated that increased infiltration of CD45RO + T cells is linked to improved OS and disease-free survival (DFS) in cancers such as nasopharyngeal carcinoma and colorectal cancer [14, 54]. Our findings of elevated *PTPRC* (CD45) expression in HNSC tumor tissue and its association with favorable survival are corroborated by data from previous study on its prognostic value [11]. However, the HPA reported negative protein expression in both normal and malignant epithelial cells. This divergence reflects the differential biological target of the two platforms. Because *PTPRC* is a pan-leukocyte marker, the elevated RNA levels capture dense immune cell infiltration within the whole tumor mass, rather than expression by carcinoma cells. Conversely, HPA’s immunohistochemistry focuses on epithelial parenchymal cells, where *PTPRC* is expectedly absent. Thus, *PTPRC* acts as a microenvironment-derived prognostic biomarker rather than an oncogenic tumor-cell marker. These findings reinforce the relevance of immune cell-derived markers in the characterization of the TME and their prognostic implications in OSCC.

The integration of miRNA–TF–gene regulatory networks via miRNet highlighted upstream regulatory complexity, with 13 miRNAs and 69 TFs influencing hub gene expression. Such multi-layered regulation provides a mechanistic basis for heterogeneity and offers potential therapeutic entry points.

## Conclusion

This integrative bioinformatics approach successfully identified five hub genes, *EGFR*, *FN1*, *IL6*, *STAT1*, and *PTPRC*, that are characterized in OSCC across five independent transcriptomic datasets. These genes reflect the complex molecular and immunological heterogeneity of OSCC and demonstrate strong associations with immune infiltration patterns and patient survival outcomes. Although *EGFR* and *FN1* may serve as potential hub genesand prognostic indicators, *PTPRC* emerges as a favorable microenvironment-derived immune marker.. The dual role of *STAT1* and the stromal-derived expression of *IL6* underscore the need for context-dependent interpretation of biomarkers. Together, these *in-silico* results provide a systems-level insight into OSCC biology and highlight potential molecular targets for future diagnostic and therapeutic strategies.

Despite the comprehensive large scale transcriptomic study, some limitations should be acknowledged. The datasets included in the study represented diverse clinical conditions, including risk factors such as alcohol/tobacco use, metastatic, recurrent, and potentially HPV-associated OSCC cases; however, clinical factors were not uniformly available across all the datasets, restricting risk factor-specific analyses. Survival and immune infiltration analyses were based on the TCGA-HNSC cohort, which includes non-oral cancers, possibly reducing OSCC specificity. Finally, the lack of experimental validation warrants further functional and clinical studies to confirm the biological relevance of the identified biomarkers.

## Supplementary Information

Supplementary Material 1.

## Data Availability

All data supporting the findings of this study are available within the paper and its Supplementary Information.

## Glossary

BaRC: Bioinformatics and Research Computing
BP: Biological Process
CC: Cellular Component
CC: Clustering Coefficient
CI: Confidence Interval
DAVID: Database for Annotation, Visualization and Integrated Discovery
DMNC: Density of Maximum Neighborhood Component
DEGs: Differentially Expressed Genes
DFS: Disease-Free Survival
EPC: Edge Percolated Component
*EGFR*: Epidermal Growth Factor Receptor
FN1: Fibronectin 1
GEO: Gene Expression Omnibus
GEPIA: Gene Expression Profiling Interactive Analysis
GO: Gene Ontology
Gtex: Genotype Tissue Expression
HR: Hazard Ratio
HNSC: Head and Neck Squamous Cancer
HPV: Human Papillomavirus
*IL6*: Interleukin 6
ITH: Intra-Tumoral Heterogeneity
KEGG: Kyoto Encyclopedia of Genes and Genomes
MCC: Maximal Clique Centrality
MNC: Maximum Neighborhood Component
MSCs: Mesenchymal Stem Cells
MCODE: Molecular Complex Detection
MF: Molecular Functions
NCBI: National Center for Biotechnology Information
OSCC: Oral Squamous Cell Carcinoma
OS: Overall Survival
PTP: Protein Tyrosine Phosphatase
PTPRC: Protein Tyrosine Phosphatase Receptor Type C
PPI: Protein-Protein Interaction
STRING: Search Tool for the Retrieval of INteracting Genes
*STAT1*: Signal Transducer and Activator of Transcription 1
TCGA: The Cancer Genome Atlas
HPA: The Human Protein Atlas
TF: Transcription Facto
TIMER: Tumor Immune Estimation Resource
TME: Tumor Microenvironment
