# Correction: Identification of potential biomarkers for oral squamous cell carcinoma through multi-cohort bioinformatic analysis

**DOI:** 10.1186/s43046-026-00413-3

**Published:** 2026-09-16

**Authors:** Duanreiliu Kamei, Simran Kaur, Anjali Priya, Akshay Bansal, Aarti Yadav, Ashwini Kumar Ray, Yamini Agrawal

**Affiliations:** 1 Department of Botany, University of Delhi, New Delhi, India; 2 Department of Environmental Studies, University of Delhi, New Delhi, India; 3 Department of Periodontology, Inderprastha Dental College & Hospital, Ghaziabad, India; 4 Lady Irwin College, University of Delhi, New Delhi, India

**Correction: J Egypt Natl Canc Inst 38, 56 (2026)**

**https://doi.org/10.1186/s43046-026-00392-5**

Following publication of the original article [1], the authors identified an error in the author name of Ashwini Kumar Ray and in the Authors’ Contributions section.

The incorrect author name is: Ashwini Ray.

The correct author name is: Ashwini Kumar Ray.

The Authors’ Contributions currently reads:

The work was designed and conceptualized by YA and AY. DK and SK analyzed the data and performed the work. The manuscript was prepared and written by DK and SK. AP provided on the development of the methodology pipeline. AKR and AKB assisted in proofreading the manuscript. All authors read and approved the final manuscript.

The Authors’ Contributions should read:

Conceptualized and designed the study: YA, AY and AKR. Development of methodology pipeline: AP, AKR and DK. Acquisition and interpretation of the data: DK, AP and SK. Writing- Original Draft preparation: DK and SK. Writing- Review and Editing: AP, AKR, YA and AY. Proofreading and Validation: AKB and AKR. Supervision: AKR, YA and AY. All authors read and approved the final manuscript.

The author group has been updated above and the original article [1] has been corrected.
