# Supplementary material for: Identification of potential biomarkers for oral squamous cell carcinoma through multi-cohort bioinformatic analysis
Source: J Egypt Natl Canc Inst. 2026 Aug 11;38:56. doi: 10.1186/s43046-026-00392-5 (PMC13462001; doi:10.1186/s43046-026-00392-5)
Supplement: Supplementary file 1 [file 43046_2026_392_MOESM1_ESM.docx]

**Supplementary Figure S1:** Top 3 modules of OSCC having MCODE score > 10: (a) Module 1 with an MCODE score of 37.837 had 50 nodes and 927 edges, including hub genes PTPRC and STAT1. (b) Module 2 with an MCODE score of 29.88 had 84 nodes and 1240 edges, including hub genes IL6 and FN1, and (c) Module 3 with an MCODE score of 18.355 had 108 nodes and 982 edges, including EGFR.

b)

a)

c)

**Supplementary Table S1:** OSCC datasets used in the study

| **Microarray datasets** | **Control** | **Cases** | **Platforms** |
| --- | --- | --- | --- |
| GSE30784 | 45 | 167 | [GPL570](https://www.ncbi.nlm.nih.gov/geo/query/acc.cgi?acc=GPL570) |
| GSE37991 | 40 | 40 | [GPL6883](https://www.ncbi.nlm.nih.gov/geo/query/acc.cgi?acc=GPL6883) |
| GSE25099 | 22 | 57 | [GPL5175](https://www.ncbi.nlm.nih.gov/geo/query/acc.cgi?acc=GPL5175) |
| GSE23558 | 5 | 27 | [GPL6480](https://www.ncbi.nlm.nih.gov/geo/query/acc.cgi?acc=GPL6480) |
| GSE70604 | 1 | 7 | [GPL2986](https://www.ncbi.nlm.nih.gov/geo/query/acc.cgi?acc=GPL2986) |

**Supplementary Table S2:** Top 20 genes ranked by network topology measures; a) degree; b) EPC; c) bottleneck; d) betweenness; e) closeness

| **Gene symbol** | **Gene name** | **Degree** | **Clustering coefficient** |
| --- | --- | --- | --- |
| *IL6* | Interleukin 6 | 307 | 0.15554 |
| IL1B | Interleukin 1 Beta | 275 | 0.17465 |
| *EGFR* | Epidermal Growth Factor Receptor | 264 | 0.12444 |
| *FN1* | Fibronectin 1 | 254 | 0.15689 |
| TGFB1 | Transforming Growth Factor Beta 1 | 205 | 0.23788 |
| CXCL8 | C-X-C Motif Chemokine Ligand 8 (also known as IL-8) | 204 | 0.23892 |
| MMP9 | Matrix Metallopeptidase 9 | 203 | 0.23821 |
| *PTPRC* | Protein Tyrosine Phosphatase Receptor Type C (CD45) | 198 | 0.25083 |
| ICAM1 | Intercellular Adhesion Molecule 1 | 184 | 0.2756 |
| CDH1 | Cadherin 1 (E-cadherin) | 172 | 0.16735 |
| IL1A | Interleukin 1 Alpha | 165 | 0.31183 |
| CXCL10 | C-X-C Motif Chemokine Ligand 10 | 164 | 0.31647 |
| CXCR4 | C-X-C Motif Chemokine Receptor 4 | 156 | 0.19198 |
| ERBB2 | Erb-B2 Receptor Tyrosine Kinase 2 (HER2) | 156 | 0.3426 |
| CSF2 | Colony Stimulating Factor 2 (Granulocyte-Macrophage) | 153 | 0.34512 |
| CXCL12 | C-X-C Motif Chemokine Ligand 12 | 153 | 0.35131 |
| JUN | Jun Proto-Oncogene, AP-1 Transcription Factor Subunit | 152 | 0.21941 |
| *STAT1* | Signal Transducer and Activator of Transcription 1 | 152 | 0.28442 |
| PXDN | Peroxidasin | 149 | 0.25503 |
| COL1A1 | Collagen Type I Alpha 1 Chain | 149 | 0.23345 |

**b)**

| **Gene symbol** | **Gene name** | **EPC** | **Clustering coefficient** |
| --- | --- | --- | --- |
| *IL6* | Interleukin 6 | 173.027 | 0.15554 |
| IL1B | Interleukin 1 Beta | 167.314 | 0.17465 |
| TGFB1 | Transforming Growth Factor Beta 1 | 164.365 | 0.23788 |
| *FN1* | Fibronectin 1 | 162.152 | 0.15689 |
| MMP9 | Matrix Metallopeptidase 9 | 162.067 | 0.23821 |
| CXCL8 | C-X-C Motif Chemokine Ligand 8 | 161.822 | 0.23892 |
| *PTPRC* | Protein Tyrosine Phosphatase Receptor Type C | 158.394 | 0.25083 |
| ICAM1 | Intercellular Adhesion Molecule 1 | 152.863 | 0.2756 |
| IL1A | Interleukin 1 alpha | 152.685 | 0.31183 |
| CXCR4 | C-X-C Motif Chemokine Receptor 4 | 151.942 | 0.3426 |
| CSF2 | Colony Stimulating Factor 2 | 151.61 | 0.35131 |
| *EGFR* | Epidermal Growth Factor Receptor | 151.325 | 0.12444 |
| CXCL12 | C-X-C Motif Chemokine Ligand 12 | 148.462 | 0.34512 |
| CXCL10 | C-X-C Motif Chemokine Ligand 10 | 148.272 | 0.31647 |
| CD86 | Cluster of Differentiation 86 | 144.462 | 0.3621 |
| TLR2 | Toll-Like Receptor 2 | 142.739 | 0.34779 |
| CTLA4 | Cytotoxic T-Lymphocyte Associated Protein 4 | 141.971 | 0.41102 |
| PXDN | Peroxidasin | 141.685 | 0.25503 |
| *STAT1* | Signal Transducer and Activator of Transcription 1 | 141.236 | 0.28442 |
| CD19 | Cluster of Differentiation 19 | 140.135 | 0.31025 |

**c)**

| **Gene symbol** | **Gene name** | **Bottleneck** | **clustering coefficient** |
| --- | --- | --- | --- |
| *IL6* | Interleukin 6 | 46 | 0.15554 |
| *EGFR* | Epidermal Growth Factor Receptor | 33 | 0.12444 |
| CALML5 | Calmodulin Like 5 | 30 | 0.10476 |
| *FN1* | Fibronectin 1 | 26 | 0.15689 |
| MUC1 | Mucin 1, Cell Surface Associated | 21 | 0.21819 |
| CD2 | CD2 Molecule | 21 | 0.38316 |
| FOS | Fos Proto-Oncogene, AP-1 Transcription Factor Subunit | 18 | 0.22109 |
| JUN | Jun Proto-Oncogene, AP-1 Transcription Factor Subunit | 16 | 0.21941 |
| SHC1 | SHC Adaptor Protein 1 | 16 | 0.2694 |
| ERBB2 | Erb-B2 Receptor Tyrosine Kinase 2 | 14 | 0.19198 |
| CDH1 | Cadherin 1 (E-Cadherin) | 14 | 0.16735 |
| TYR | Tyrosinase | 14 | 0.28333 |
| *PTPRC* | Protein Tyrosine Phosphatase Receptor Type C | 13 | 0.25083 |
| BMP7 | Bone Morphogenetic Protein 7 | 13 | 0.29847 |
| OCLN | Occludin | 12 | 0.27163 |
| *STAT1* | Signal Transducer and Activator of Transcription 1 | 11 | 0.28442 |
| CDKN2A | Cyclin Dependent Kinase Inhibitor 2A | 11 | 0.26323 |
| NGF | Nerve Growth Factor | 11 | 0.34024 |
| PLEK | Pleckstrin | 11 | 0.14787 |
| ICAM1 | Intercellular Adhesion Molecule 1 | 10 | 0.2756 |

**d)**

| **Gene symbol** | **Gene name** | **Betweenness** | **Clustering coefficient** |
| --- | --- | --- | --- |
| *EGFR* | Epidermal Growth Factor Receptor | 167232.4815 | 0.12444 |
| *IL6* | Interleukin 6 | 121381.9424 | 0.15554 |
| *FN1* | Fibronectin 1 | 99631.31539 | 0.15689 |
| CDH1 | Cadherin 1 (E-Cadherin) | 75738.65104 | 0.16735 |
| IL1B | Interleukin 1 Beta | 73920.88346 | 0.17465 |
| ERBB2 | Erb-B2 Receptor Tyrosine Kinase 2 | 55371.25744 | 0.19198 |
| CALML5 | Calmodulin Like 5 | 54572.19098 | 0.10476 |
| MUC1 | Mucin 1, Cell Surface Associated | 49792.54811 | 0.21819 |
| *PTPRC* | Protein Tyrosine Phosphatase Receptor Type C | 46384.61651 | 0.25083 |
| CAV1 | Caveolin 1 | 45648.42699 | 0.20082 |
| JUN | Jun Proto-Oncogene, AP-1 Transcription Factor Subunit | 42953.56294 | 0.21941 |
| PPARG | Peroxisome Proliferator Activated Receptor Gamma | 38844.52319 | 0.22971 |
| MMP9 | Matrix Metallopeptidase 9 | 35044.89285 | 0.23821 |
| TGFB1 | Transforming Growth Factor Beta 1 | 32925.68392 | 0.23788 |
| PXDN | Peroxidasin | 32445.7171 | 0.25503 |
| *STAT1* | Signal Transducer and Activator of Transcription 1 | 32152.86112 | 0.28442 |
| KRT5 | Keratin 5 | 30696.05269 | 0.18607 |
| CALML6 | Calmodulin Like 6 | 30121.27518 | 0.12909 |
| FYN | FYN Proto-Oncogene, Src Family Tyrosine Kinase | 29697.44479 | 0.22464 |
| CXCL8 | C-X-C Motif Chemokine Ligand 8 (also known as IL-8) | 28543.49922 | 0.23892 |

**e)**

| **Gene symbol** | **Gene name** | **Closeness** | **clustering coefficient** |
| --- | --- | --- | --- |
| *IL6* | Interleukin 6 | 859.68333 | 0.15554 |
| *EGFR* | Epidermal Growth Factor Receptor | 843.18333 | 0.12444 |
| IL1B | Interleukin 1 Beta | 834.68333 | 0.17465 |
| *FN1* | Fibronectin 1 | 827.7 | 0.15689 |
| TGFB1 | Transforming Growth Factor Beta 1 | 788.25 | 0.23788 |
| MMP9 | Matrix Metallopeptidase 9 | 786.68333 | 0.23821 |
| CXCL8 | C-X-C Motif Chemokine Ligand 8 (Interleukin-8) | 782.86667 | 0.23892 |
| *PTPRC* | Protein Tyrosine Phosphatase Receptor Type C | 781.48333 | 0.25083 |
| CDH1 | Cadherin 1 (E-Cadherin) | 777.18333 | 0.16735 |
| ICAM1 | Intercellular Adhesion Molecule 1 | 767.18333 | 0.2756 |
| ERBB2 | Erb-B2 Receptor Tyrosine Kinase 2 | 766.6 | 0.19198 |
| PXDN | Peroxidasin | 761.85 | 0.25503 |
| JUN | Jun Proto-Oncogene, AP-1 Transcription Factor Subunit | 760.03333 | 0.21941 |
| CXCR4 | C-X-C Motif Chemokine Receptor 4 | 755.43333 | 0.3426 |
| IL1A | Interleukin 1 Alpha | 753.83333 | 0.31183 |
| CXCL12 | C-X-C Motif Chemokine Ligand 12 | 744.38333 | 0.34512 |
| *STAT1* | Signal Transducer and Activator of Transcription 1 | 740.73333 | 0.28442 |
| COL1A1 | Collagen Type I Alpha 1 Chain | 739.63333 | 0.23345 |
| PPARG | Peroxisome Proliferator Activated Receptor Gamma | 738.73333 | 0.22971 |
| PTGS2 | Prostaglandin-Endoperoxide Synthase 2 (COX-2) | 736.33333 | 0.28763 |

**Supplementary Table S3:** Immunohistochemical profiling of selected hub genes in normal and HNSC Tissues.

| **Gene** | ***EGFR*** | | ***FN1*** | | ***IL6*** | | ***STAT1*** | | ***PTPRC*** | |
| --- | --- | --- | --- | --- | --- | --- | --- | --- | --- | --- |
| **Antibody** | HPA018530 | | HPA027066 | | CAB023406 | | HPA000931 | | HPA000440 | |
| **Tissue** | Normal | HNSC | Normal | HNSC | Normal | HNSC | Normal | HNSC | Normal | HNSC |
| **Staining** | High | High | Low | Low | Low | Not detected | Medium | Medium | Not detected | Not detected |
| **Intensity** | High | High | Weak | Moderate | Moderate | Negative | Moderate | Moderate | Negative | Negative |

**Supplementary Table S4:** Correlation between *EGFR*, *FN1*, *IL6*, *STAT1*, and *PTPRC* gene expression and immune cell infiltrating level derived from the TIMER 2.0 database.

| Hub genes  Immune cell | **Partial correlation** | | | | | | |
| --- | --- | --- | --- | --- | --- | --- | --- |
|  | Purity | B Cells | CD8+ cells | CD4+ cells | Macrophages | Neutrophils | Dendritic cells |
| *EGFR* | 0.03 | -0.008 | -0.105 | 0.24 | 0.05 | 0.067 | 0.132 |
| *FN1* | -0.09 | 0.138 | 0.017 | 0.387 | 0.457 | 0.229 | 0.394 |
| *IL6* | -0.157 | -0.01 | 0.109 | 0.078 | 0.162 | 0.176 | 0.18 |
| *STAT1* | -0.24 | 0.038 | 0.328 | 0.514 | 0.277 | 0.761 | 0.555 |
| *PTPRC* | -0.307 | 0.463 | 0.667 | 0.735 | 0.701 | 0.757 | 0.884 |
